# MBECS: Microbiome Batch Effects Correction Suite

**DOI:** 10.1186/s12859-023-05252-w

**Published:** 2023-05-03

**Authors:** Michael Olbrich, Axel Künstner, Hauke Busch

**Affiliations:** 1 Lübeck Institute for Experimental Dermatology, University of Lübeck, Lübeck, Germany; 2 Institute for Cardiogenetics, University of Lübeck, Lübeck, Germany; 3 Center for Biotechnology, Khalifa University, Abu Dhabi, United Arab Emirates

**Keywords:** Microbiome, Batch effects, R-package, phyloseq, Bioconductor

## Abstract

Despite the availability of batch effect correcting algorithms (BECA), no comprehensive tool that combines batch correction and evaluation of the results exists for microbiome datasets. This work outlines the Microbiome Batch Effects Correction Suite development that integrates several BECAs and evaluation metrics into a software package for the statistical computation framework R.

**Supplementary Information:**

The online version contains supplementary material available at 10.1186/s12859-023-05252-w.

## Introduction

The emergence of unwanted variation in next-generation sequencing applications is a well-researched challenge. A particular form of unwanted technical variation are batch effects (BE) that potentially result from any distinct grouping of samples during the processing steps. Hence, the introduced variability reflects the differences in, for example, the environmental conditions, batches of reagents, sequencing machines, or sample handling for corresponding batches [1, 2]. Consequently, unwanted variation can negatively affect the downstream statistical analyses as it represents a confounding factor that can obscure or exacerbate the biological truth in a dataset [3]. The comprehensive scientific research into causes and strategies for preventing and correcting batch effects indicates this topic's importance [4, 5]. While appropriate measures during the planning and execution of an experiment can limit the emergence and magnitude of batch effects, they are not entirely preventable and thus need to be accounted for before statistical analyses [6]. Despite the availability of batch effect correcting algorithms (BECA) and instructive guides on mitigating of BEs [4], no comprehensive tool that combines batch correction and evaluation of the results exists for microbiome datasets. This work introduces the Microbiome Batch Effects Correction Suite (MBECS), which integrates several established BECAs and evaluation metrics into a software package for the R statistical computation framework.

## Features

The Microbiome Batch Effect Correction Suite is designed as a software toolbox that enables users to estimate the severity of batch effects, facilitates the utilization of different BECAs, and finally provides comparative metrics to evaluate the success of each method. To that end, the package offers a convenient 5-step workflow that produces a report to guide the user in selecting the optimal results for downstream analyses.

The software builds upon the phyloseq [7] package, which facilitates the intuitive import and export of existing microbiome datasets and enables the use of other count-based datasets. The packages' data object extends the phyloseq class with additional fields that store normalized and batch-corrected feature abundance tables. All operations are performed on this single data object that keeps track of the results, promoting tidy scripts and enabling MBECS comparative reporting.

The normalization methods implemented in MBECS are total-sum scaling (TSS) and centered log-ratio transformation (CLR) [8]. Available BECAs include established correction algorithms such as ComBat and Remove Batch Effects from the SVA package [9] and Remove Unwanted Variation 3 implemented in the RUV package [10]. Additionally, the package implements batch mean centering, Percentile Normalization, and Singular Value Decomposition as correction approaches [11].

Quantifying the variability in a dataset that can be attributed to batch effects is not trivial. A relative log expression (RLE) plot, for example, can indicate the presence of batch effects, yet it is not a suitable approach to determining whether or not they have been removed successfully by a correction algorithm [12]. Thus, the suite implements several distinct metrics to provide the user with comprehensive information to assess the severity of BEs before and after batch-correction procedures. Available methods include constructing linear models from recorded biological and batch factors to estimate the variability attributed to batch effects before and after the correction procedures. Further approaches implemented are partial redundancy analysis and principal variance components analysis [13, 14]. Finally, the silhouette coefficient is a qualitative measure of the goodness of fit of samples to their respective biological groupings [15].

**Fig. 1:**
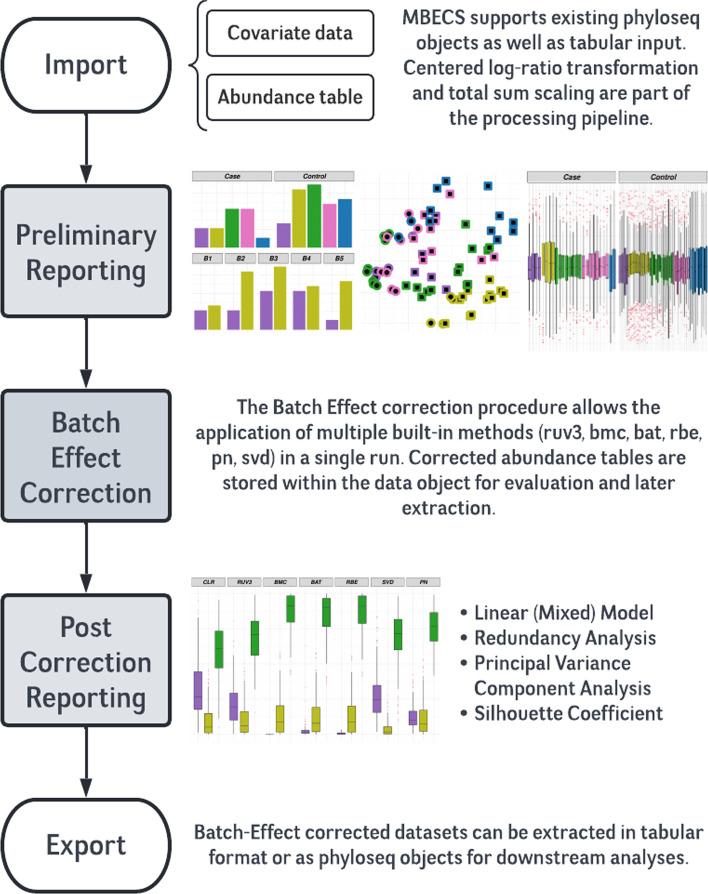
The MBECS processing pipeline comprises five main steps that provide users with the means to investigate potential batch effects and mitigate them before downstream statistical analyses: The preliminary report provides an overview of a data set to assess the presence and gravity of batch effects. The correction step can apply and store the output of various BECAs. The available correction methods are Remove Unwanted Variation 3 (ruv3), Batch Mean Centering (bmc), ComBat (bat), RemoveBatchEffects (rbe), Percentile Normalization (pn), and Singular Value Decomposition (svd). Several variance assessment methods, e.g., linear modeling, principal variance component analysis, and redundancy analysis are employed in the post-processing reports to produce a comparative qualitative analysis between the uncorrected data set and the selected BECAs. The export functionality allows extraction of the transformed or corrected counts in tabular or phyloseq formats to facilitate downstream statistical analyses

The packages' native workflow depicted in Fig. 1 will create a preliminary report upon importing the dataset. This report summarizes the data concerning covariate information, distribution of samples into biological groups and known batches, heatmaps, and box plots of the most variable features concerning the batch factor and relative log-expression plots. The preliminary report also provides the metrics mentioned above to assess variability for the uncorrected data. The user can decide whether or not batch correction is required based on that account. The subsequent processing step allows the application of selected correction methods depending on the experimental design. Methods like RUV-3 specifically require technical replicates in different batches to work; Batch mean centering is only applicable to datasets that comprise two-factor biological groupings, i.e., case–control studies [10]. Therefore, it is up to the user which methods to use, and all the correction results are stored within the data object.

The third step constructs the post-correction report. This report provides comparative analyses between uncorrected data and all the employed correction algorithms. The user can use these to evaluate the correction algorithms in terms of reduced unwanted variability while preserving the biological variation that is investigated with the experimental design. An instructive manual for the package and examples of preliminary and post-corrections reports are available as supplemental material accompanying the online article (Additional file 1, Additional file 2, Additional file 3).

## Implementation

The Microbiome Batch Effect Correction Suite is available as a software package for the R programming framework at Bioconductor. The latest development version can be obtained from the GitHub repository.

### Availability and requirements

- Project name: MBECS Microbiome Batch Effect Correction Suite
- Project home page: http://www.bioconductor.org/packages/release/bioc/html/MBECS.html
- Operating system(s): Platform independent
- Programming language: R (> = 4.1)
- Other requirements: CRAN and Bioconductor packages (methods, magrittr, phyloseq, limma, lme4, lmerTest, pheatmap, rmarkdown, cluster, dplyr, ggplot2, gridExtra, ruv, sva, tibble, tidyr, vegan, stats, utils, Matrix)
- License: Artistic-2.0
- Any restrictions to use by non-academics: None

## Supplementary Information

**Additional file 1.** Vignette.

**Additional file 2.** Preliminary report.

**Additional file 3.** Post-correction report.

## Data Availability

The source code is freely available under Artistic-2.0 license at https://github.com/rmolbrich/MBECS and at https://bioconductor.org/packages/release/bioc/html/MBECS.html. The packages vignette and examples utilize artificial mockup data to illustrate workflow and execution. The package vignette and two exemplary reports are available as supplementary data.
