# Supplementary material for: MBECS: Microbiome Batch Effects Correction Suite
Source: BMC Bioinformatics. 2023 May 3;24:182. doi: 10.1186/s12859-023-05252-w (PMC10155362; doi:10.1186/s12859-023-05252-w)
Supplement: Supplementary file 1 — Additional file 1. Vignette. [file 12859_2023_5252_MOESM1_ESM.html]

MBECS introduction


# MBECS introduction

#### 6 October 2022

# 1 Introduction

The Microbiome Batch-Effect Correction Suite aims to provide a toolkit for
stringent assessment and correction of batch-effects in microbiome data sets.
To that end, the package offers wrapper-functions to summarize study-design and
data, e.g., PCA, Heatmap and Mosaic-plots, and to estimate the proportion of
variance that can be attributed to the batch effect. The `mbecsCorrection()`
function acts as a wrapper for various batch effect correcting algorithms
(BECA) and in conjunction with the aforementioned tools, it can be used to
compare the effectiveness of correction methods on particular sets of data.
All functions of this package are accessible on their own or within the
preliminary and comparative report pipelines respectively.

## Dependencies

The `MBECS` package relies on the following packages to work:

Table 1: MBECS package dependencies

  
| Package | Version | Date | Repository |
| --- | --- | --- | --- |
| phyloseq | 1.38.0 | 2019-04-23 | NULL |
| magrittr | 2.0.3 | NULL | CRAN |
| cluster | 2.1.4 | 2022-08-19 | CRAN |
| dplyr | 1.0.10 | NULL | CRAN |
| ggplot2 | 3.3.6 | NULL | CRAN |
| gridExtra | 2.3 | NULL | CRAN |
| limma | 3.50.3 | 2022-04-06 | NULL |
| lme4 | 1.1.30 | NULL | CRAN |
| lmerTest | 3.1.3 | NULL | CRAN |
| pheatmap | 1.0.12 | 2018-12-26 | CRAN |
| rmarkdown | 2.16 | NULL | CRAN |
| ruv | 0.9.7.1 | 2019-08-30 | CRAN |
| sva | 3.42.0 | NULL | NULL |
| tibble | 3.1.8 | NULL | CRAN |
| tidyr | 1.2.1 | NULL | CRAN |
| vegan | 2.6.2 | NULL | CRAN |
| methods | 4.1.2 | NULL | NULL |
| stats | 4.1.2 | NULL | NULL |
| utils | 4.1.2 | NULL | NULL |

## Installation

`MBECS` should be installed as follows:

```
if (!"BiocManager" %in% rownames(installed.packages()))
     install.packages("BiocManager")
BiocManager::install("MBECS")
```

To install the most current (but not necessarily stable) version, use the
repository on GitHub:

```
# Use the devtools package to install from a GitHub repository.
install.packages("devtools")

# This will install the MBECS package from GitHub.
devtools::install_github("rmolbrich/MBECS")
```

# 2 Workflow

The main application of this package is microbiome data. It is common practice
to use the `phyloseq` (McMurdie et al. 2021) package for analyses of this type of data.
To that end, the `MBECS` package extends the `phyloseq` class in order to
provide its functionality. The user can utilize objects of class `phyloseq` or
a `list` object that contains an abundance table as well as meta data. The
package contains a dummy data-set of artificially generated data to illustrate
this process. To start an analysis, the user requires the `mbecProcessInput()`
function.

Load the package via the `library()` function.

```
library(MBECS)
```

Use the `data()`function to load the provided mockup data-sets at this point.

```
# List object 
data(dummy.list)
# Phyloseq object
data(dummy.ps)
# MbecData object
data(dummy.mbec)
```

## 2.1 Start from abundance table

For an input that consists of an abundance table and meta-data, both tables
require sample names as either row or column names. They need to be passed in a
`list` object with the abundance matrix as first element. The
`mbecProcessInput()` function will handle the correct orientation and return an
object of class `MbecData`.

```
# The dummy-list input object comprises two matrices:
names(dummy.list)
#> [1] "cnts" "meta"
```

The optional argument `required.col` may be used to ensure that all covariate
columns that should be there are available. For the dummy-data these are
“group”, 
“batch” and “replicate”.

```
mbec.obj <- mbecProcessInput(dummy.list, 
                             required.col = c("group", "batch", "replicate"))
```

## 2.2 Start from phyloseq object

The start is the same if the data is already of class `phyloseq`. The `dummy.ps`
object contains the same data as `dummy.list`, but it is of class `phyloseq`.
Create an `MbecData` object from `phyloseq` input.

The optional argument `required.col` may be used to ensure that all covariate
columns that should be there are available. For the dummy-data these are
“group”, 
“batch” and “replicate”.

```
mbec.obj <- mbecProcessInput(dummy.ps, 
                             required.col = c("group", "batch", "replicate"))
```

## 2.3 Apply transformations

The most common normalizing transformations in microbiome analysis are total
sum scaling (TSS) and centered log-ratio transformation (CLR). Hence, the
`MBECS` package offers these two methods. The resulting matrices will be stored
in their respective `slots (tss, clr)` in the `MbecData` object, while the
original abundance table will remain unchanged.

Use `mbecTransform()` to apply total sum scaling to the data.

```
mbec.obj <- mbecTransform(mbec.obj, method = "tss")
#> Set tss-transformed counts.
```

Apply centered log-ratio transformation to the data. Due to the sparse nature
of compositional microbiome data, the parameter `offset` may be used to add a
small offset to the abundance matrix in order to facilitate the CLR
transformation.

```
mbec.obj <- mbecTransform(mbec.obj, method = "clr", offset = 0.0001)
```

## 2.4 Preliminary report

The function `mbecReportPrelim()` will provide the user with an overview of
experimental setup and the significance of the batch effect. To that end it is
required to declare the covariates that are related to batch effect and group
effect respectively. In addition it provides the option to select the abundance
table to use here. The CLR transformed abundances are the default and the
function will calculate them if they are not present in the input. Technically,
the user can start the analysis at this point because the function incorporates
the functionality of the aforementioned processing functions.

The parameter `model.vars` is a character vector with two elements. The first
denotes the covariate column that describes the batch effect and the second one
should be used for the presumed biological effect of interest, e.g., the group
effect in case/control studies. The `type` parameter selects which abundance
table is to be used “otu”,
“clr”, “tss”
.

```
mbecReportPrelim(input.obj=mbec.obj, model.vars=c("batch","group"), 
                 type="clr")
```

## 2.5 Run corrections

The package acts as a wrapper for six different batch effect correcting
algorithms (BECA).

- Remove Unwanted Variation 3 (`ruv3`)

  - This algorithm is implemented in ruv-package by Gagnon-Bartsch (2019).
  - The algorithm requires negative control-features, i.e., features that
    are known to be unaffected by the batch effect, as well as technical
    replicates. The algorithm will check for the existence of a replicate
    column in the covariate data. If the column is not present, the execution
    stops and a warning message will be displayed. The denominators for
    negative controls can be supplied via the parameter ‘nc.features’. If they
    are not supplied, the function will employ a linear model to determine
    pseudo negative controls that are not significantly affected by the batch
    effect.
- Batch Mean Centering (`bmc`)

  - This algorithm is part of this package.
  - For known batch effects, this method takes the batches, i.e., subgroup
    of samples within a particular batch, and centers them to their mean value.
- ComBat (`bat`)

  - Described by Johnson et al. 2007 this method was initially conceived to
    work with gene expression data and is part of the sva-package by Leek et al. (2021).
  - This method uses an non-/parametric empirical Bayes framework to
    correct for known batch effects.
- Remove Batch Effect (`rbe`)

  - As part of the limma-package by Smyth et al. (2021) this method was designed to
    remove BEs from Micro array data.
  - The algorithm fits the full-model to the data, i.e., all relevant
    covariates whose effect should not be removed, and a model that only
    contains the known batch effects. The difference between these models
    produces a residual matrix that (presumably) contains only the
    full-model-effect, e.g., treatment.
- Percentile Normalization (`pn`)

  - This method was actually developed specifically to facilitate the
    integration of microbiome data from different studies/experimental set-ups
    by Gibbons, Duvallet, and Alm (2018). This problem is similar to the mitigation of BEs, i.e.,
    when collectively analyzing two or more data-sets, every study is
    effectively a batch on its own (not withstanding the probable BEs within
    studies).
  - The algorithm iterates over the unique batches and converts the
    relative abundance of control samples into their percentiles. The relative
    abundance of case-samples within the respective batches is then transformed
    into percentiles of the associated control-distribution. Basically, the
    procedure assumes that the control-group is unaffected by any effect of
    interest, e.g., treatment or sickness, but both groups within a batch are
    affected by that BE. The switch to percentiles (kinda) flattens the
    effective difference in count values due to batch - as compared to the
    other batches. This also introduces the two limiting aspects in percentile
    normalization. It can only be applied to case/control designs because it
    requires a reference group. In addition, the transformation into
    percentiles removes information from the data.
  - The ‘mbecRunCorrections’ wrapper will
    automatically select Total Sum Scaled values for percentile normalization
    because that is what it is supposed to be used on. The function
    `mbecCorrections` can be used to manually select untransformed or centered
    log ratio transformed abundances.
- Support Vector Decomposition (`svd`)

  - Successfully applied to micro-array data by Nielsen et al. (2002).
  - Basically perform matrix factorization and compute singular
    eigen-vectors (SEV). Assume that the first SEV captures the batch-effect
    and remove this effect from the data.

The user has the choice between two functions `mbecCorrection()` and
`mbecRunCorrections()`, the latter one acts as a wrapper that can apply multiple
correction methods in a single run. If the input
to `mbecRunCorrections()` is missing CLR and TSS transformed abundance matrices,
they will be created with default settings, i.e., offsets for zero values will
be set to 1/#features.

The function `mbecCorrection()` will apply a single correction algorithm
selected by the parameter `method` and return an object that contains the
resulting corrected abundance matrix in its `cor slot` with the respective name.

```
mbec.obj <- mbecCorrection(mbec.obj, model.vars=c("batch","group"), 
                           method = "bat", type = "clr")
#> Applying ComBat (sva) for batch-correction.
#> Found2batches
#> Adjusting for1covariate(s) or covariate level(s)
#> Standardizing Data across genes
#> Fitting L/S model and finding priors
#> Finding nonparametric adjustments
#> Adjusting the Data
```

The function `mbecRunCorrections()` will apply all correction algorithms
selected by the parameter `method` and return an object that contains all
respective corrected abundance matrices in the `cor` slot. In this example
there will be three in total, named like the methods that created them.

```
mbec.obj <- mbecRunCorrections(mbec.obj, model.vars=c("batch","group"),
                               method=c("ruv3","rbe","bmc","pn","svd"), 
                               type = "clr")
#> No negative control features provided.
#>                 Using pseudo-negative controls.
#> Applying Remove Unwanted Variantion v3 (RUV-III).
#> Applying 'removeBatchEffect' (limma) for batch-correction.
#> Applying Batch Mean-Centering (BMC) for batch-correction.
#> Applying Percentile Normalization (PN).
#> Warning in mbecPN(input.obj, model.vars, type = eval(type)): Abundances contain
#> zero values. Adding small uniform offset.
#> Group A is considered control group, i.e., reference
#>           for normalization procedure. To change reference please 'relevel()'
#>           grouping factor accordingly.
#> Applying Singular Value Decomposition (SVD) for batch-correction.
```

## 2.6 Post report

The `mbecReportPost()` function will provide the user with a comparative report
that shows how the chosen batch effect correction algorithms changed the
data-set compared to the initial values.

The parameter `model.vars` is a character vector with two elements. The first
denotes the covariate column that describes the batch effect and the second one
should be used for the presumed biological effect of interest, e.g., the group
effect in case/control studies. The `type` parameter selects which abundance
table is to be used “otu”,
“clr”, “tss”
.

```
mbecReportPost(input.obj=mbec.obj, model.vars=c("batch","group"), 
               type="clr")
```

## 2.7 Retrieve corrrected data

Because the `MbecData` class extends the `phyloseq` class, all functions from
`phyloseq` can be used as well. They do however only apply to the `otu_table`
slot and will return an object of class `phyloseq`, i.e., any transformations
or corrections will be lost. To retrieve an object of class `phyloseq` that
contains the `otu_table` of corrected counts, for downstream analyses, the user
can employ the `mbecGetPhyloseq()` function. As before, the arguments `type` and
`label` are used to specify which abundance table should be used in the
returned object.

To retrieve the CLR transformed counts, set `type` accordingly.

```
ps.clr <- mbecGetPhyloseq(mbec.obj, type="clr")
```

If the batch-mean-centering corrected counts show the best results, select
“cor” as `type` and set the `label` to
“bmc”.

```
ps.bmc <- mbecGetPhyloseq(mbec.obj, type="cor", label="bmc")
```

# 3 Use single functions

## 3.1 Exploratory Functions

### 3.1.1 Relative Log-Expression

Relative Log-Expression plots can be created with the `mbecRLE()` function.

Produce RLE-plot on CLR transformed values. The `return.data` parameter can be
set to TRUE to retrieve the data for
inspection or use in your own plotting function.

```
rle.plot <- mbecRLE(input.obj=mbec.obj, model.vars = c("batch","group"), 
                    type="clr",return.data = FALSE) 
#> Calculating RLE for group: A
#> Calculating RLE for group: B

# Take a look.
plot(rle.plot)
```

### 3.1.2 Principal Components Analysis

PCA plots can be created with the `mbecPCA()` function.

Produce PCA-plot on CLR transformed values. The principal components can be
selected with the `pca.axes` parameter. The vector of length two works like
this c(x-axis,
y-axis). The return data parameter can
be set to TRUE to retrieve the data for
inspection or use in your own plotting function.

```
pca.plot <- mbecPCA(input.obj=mbec.obj, model.vars = "group", type="clr",
                    pca.axes=c(1,2), return.data = FALSE)
```

Plot with two grouping variables, determining color and shape of the output.

```
pca.plot <- mbecPCA(input.obj=mbec.obj, model.vars = c("batch","group"),
                    type="clr", pca.axes=c(1,2), return.data = FALSE)
```

### 3.1.3 Box Plot

Box plots of the most variable features can be create with the `mbecBox()`
function.

Produce Box-plots of the most variable features on CLR transformed values. The
`method` parameter determines if “ALL” or
only the “TOP” n features are plotted. The
`n` parameter selects the number of features to plot. The function will return a
`list` of plot objects to be used.

```
box.plot <- mbecBox(input.obj=mbec.obj, method = "TOP", n = 2, 
                    model.var = "batch", type="clr", return.data = FALSE) 

# Take a look.
plot(box.plot$OTU109)
```

Setting `method` to a vector of feature names will select exactly these
features for plotting.

```
box.plot <- mbecBox(input.obj=mbec.obj, method = c("OTU1","OTU2"), 
                    model.var = "batch", type="clr", return.data = FALSE) 
#> 'Method' parameter contains multiple elements -
#>             using to select features.

# Take a look.
plot(box.plot$OTU2)
```

### 3.1.4 Heatmap

Pheatmap is used to produce heat-maps of the most variable features with the
`mbecHeat()` function.

Produce a heatmap of the most variable features on CLR transformed values. The
`method` parameter determines if “ALL” or
only the “TOP” n features are plotted. The
`n` parameter selects the number of features to plot. The parameters `center`
and `scale` can be used to de-/activate centering and scaling prior to plotting.
The `model.vars` parameter denotes all covariates of interest.

```
heat.plot <- mbecHeat(input.obj=mbec.obj, method = "TOP", n = 10, 
                    model.vars = c("batch","group"), center = TRUE,
                     scale = TRUE, type="clr", return.data = FALSE)
```

### 3.1.5 Mosaic

A mosaic plot of the distribution of samples over two covariates of interest
can be produced with the `mbecMosaic()` function.

```
mosaic.plot <- mbecMosaic(input.obj = mbec.obj, 
                          model.vars = c("batch","group"),
                          return.data = FALSE)
```

## 3.2 Analysis of Variance

The functions aim to determine the amount of variance that can be attributed to
selected covariates of interest and visualize the results.

### 3.2.1 Linear Model

Use the function `mbecModelVariance()` with parameter `method` set to
“lm” to fit a linear model of the form
`y ~ group + batch` to every feature. Covariates of interest can be selected
with the `model.vars` parameter and the function will construct a
model-formula. The parameters `type` and `label` can be used to select the
desired abundance matrix to work on This defaults to CLR transformed values.

Plot the resulting data with the `mbecVarianceStatsPlot()` function and show the
proportion of variance with regards to the covariates in a box-plot.

```
lm.variance <- mbecModelVariance(input.obj=mbec.obj, 
                                 model.vars = c("batch", "group"),
                                 method="lm",type="cor", label="svd")

# Produce plot from data.
lm.plot <- mbecVarianceStatsPlot(lm.variance)

# Take a look.
plot(lm.plot)
```

### 3.2.2 Linear Mixed Model

Use the function `mbecModelVariance()` with parameter `method` set to
“lmm” to fit a linear mixed model of the
form `y ~ group + (1|batch)` to every feature. Covariates of interest can be
selected with the `model.vars` parameter and the function will construct a
model-formula. The parameters `type` and `label` can be used to select the
desired abundance matrix to work on This defaults to CLR transformed values.

Plot the resulting data with the `mbecVarianceStatsPlot()` function and show the
proportion of variance with regards to the covariates in a box-plot.

```
lmm.variance <- mbecModelVariance(input.obj=mbec.obj, 
                                  model.vars = c("batch","group"), 
                                  method="lmm",
                                  type="cor", label="ruv3")

# Produce plot from data.
lmm.plot <- mbecVarianceStatsPlot(lmm.variance)

# Take a look.
plot(lmm.plot)
```

### 3.2.3 Redundancy Analysis

Use the function `mbecModelVariance()` with parameter `method` set to
“rda” to calculate the percentage of
variance that can be attributed to the covariates of interest with partial
Redundancy Analysis. Covariates of interest can be selected with the
`model.vars` parameter. The parameters `type` and `label` can be used to select
the desired abundance matrix to work on This defaults to CLR transformed values.

Plot the resulting data with the `mbecRDAStatsPlot()` function and show the
percentage of variance with regards to the covariates in a bar-plot.

```
rda.variance <- mbecModelVariance(input.obj=mbec.obj, 
                                  model.vars = c("batch", "group"),
                                  method="rda",type="cor", label="bat")

# Produce plot from data.
rda.plot <- mbecRDAStatsPlot(rda.variance)

# Take a look.
plot(rda.plot)
```

### 3.2.4 Principal Variance Components Analysis

Use the function `mbecModelVariance()` with parameter `method` set to
“pvca” to calculate the percentage of
variance that can be attributed to the covariates of interest using Principal
Variance Components Analysis. Covariates of interest can be selected with the
`model.vars` parameter. The parameters `type` and `label` can be used to select
the desired abundance matrix to work on This defaults to CLR transformed values.

Plot the resulting data with the `mbecPVCAStatsPlot()` function and show the
percentage of variance with regards to the covariates in a bar-plot.

```
pvca.variance <- mbecModelVariance(input.obj=mbec.obj, 
                                   model.vars = c("batch", "group"),
                                   method="pvca",type="cor", label="rbe")

# Produce plot from data.
pvca.plot <- mbecPVCAStatsPlot(pvca.variance)

# Take a look.
plot(pvca.plot)
```

### 3.2.5 Silhouette Coefficient

Evaluate how good the samples fit to the clustering that is implied by the
covariates of interest.

Use the function `mbecModelVariance()` with parameter `method` set to
“s.coef” to evaluate the clustering that is
implied by the covariates of interest with the Silhouette Coefficient.
Covariates of interest can be selected with the `model.vars` parameter. The
parameters `type` and `label` can be used to select the desired abundance
matrix to work on This defaults to CLR transformed values.

Plot the resulting data with the `mbecSCOEFStatsPlot()` function and show the
silhouette coefficients in a dot-plot.

```
sil.coefficient <- mbecModelVariance(input.obj=mbec.obj, 
                                     model.vars = c("batch", "group"),
                                     method="s.coef",type="cor", label="bmc")

# Produce plot from data.
scoef.plot <- mbecSCOEFStatsPlot(sil.coefficient)

# Take a look.
plot(scoef.plot)
```

# 4 Session

```
#> R version 4.1.2 (2021-11-01)
#> Platform: x86_64-apple-darwin17.0 (64-bit)
#> Running under: macOS Big Sur 10.16
#> 
#> Matrix products: default
#> BLAS:   /Library/Frameworks/R.framework/Versions/4.1/Resources/lib/libRblas.0.dylib
#> LAPACK: /Library/Frameworks/R.framework/Versions/4.1/Resources/lib/libRlapack.dylib
#> 
#> attached base packages:
#> [1] stats     graphics  grDevices utils     datasets  methods   base     
#> 
#> other attached packages:
#> [1] MBECS_1.1.0      BiocStyle_2.22.0
#> 
#> loaded via a namespace (and not attached):
#>   [1] minqa_1.2.4            colorspace_2.0-3       ellipsis_0.3.2        
#>   [4] XVector_0.34.0         rstudioapi_0.14        farver_2.1.1          
#>   [7] bit64_4.0.5            AnnotationDbi_1.56.2   fansi_1.0.3           
#>  [10] codetools_0.2-18       splines_4.1.2          cachem_1.0.6          
#>  [13] knitr_1.40             ade4_1.7-19            jsonlite_1.8.0        
#>  [16] phyloseq_1.38.0        nloptr_2.0.3           annotate_1.72.0       
#>  [19] cluster_2.1.4          png_0.1-7              pheatmap_1.0.12       
#>  [22] BiocManager_1.30.18    compiler_4.1.2         httr_1.4.4            
#>  [25] assertthat_0.2.1       Matrix_1.4-1           fastmap_1.1.0         
#>  [28] limma_3.50.3           cli_3.4.0              htmltools_0.5.3       
#>  [31] tools_4.1.2            igraph_1.3.4           gtable_0.3.1          
#>  [34] glue_1.6.2             GenomeInfoDbData_1.2.7 reshape2_1.4.4        
#>  [37] dplyr_1.0.10           Rcpp_1.0.9             Biobase_2.54.0        
#>  [40] jquerylib_0.1.4        vctrs_0.4.1            Biostrings_2.62.0     
#>  [43] rhdf5filters_1.6.0     multtest_2.50.0        ape_5.6-2             
#>  [46] nlme_3.1-159           iterators_1.0.14       xfun_0.33             
#>  [49] stringr_1.4.1          lme4_1.1-30            lifecycle_1.0.2       
#>  [52] XML_3.99-0.10          edgeR_3.36.0           zlibbioc_1.40.0       
#>  [55] MASS_7.3-58.1          scales_1.2.1           parallel_4.1.2        
#>  [58] biomformat_1.22.0      rhdf5_2.38.1           RColorBrewer_1.1-3    
#>  [61] yaml_2.3.5             memoise_2.0.1          gridExtra_2.3         
#>  [64] ggplot2_3.3.6          sass_0.4.2             stringi_1.7.8         
#>  [67] RSQLite_2.2.17         highr_0.9              genefilter_1.76.0     
#>  [70] S4Vectors_0.32.4       foreach_1.5.2          permute_0.9-7         
#>  [73] BiocGenerics_0.40.0    boot_1.3-28            BiocParallel_1.28.3   
#>  [76] GenomeInfoDb_1.30.1    rlang_1.0.5            pkgconfig_2.0.3       
#>  [79] bitops_1.0-7           matrixStats_0.62.0     evaluate_0.16         
#>  [82] lattice_0.20-45        purrr_0.3.4            Rhdf5lib_1.16.0       
#>  [85] ruv_0.9.7.1            labeling_0.4.2         bit_4.0.4             
#>  [88] tidyselect_1.1.2       plyr_1.8.7             magrittr_2.0.3        
#>  [91] bookdown_0.29          R6_2.5.1               IRanges_2.28.0        
#>  [94] magick_2.7.3           generics_0.1.3         DBI_1.1.3             
#>  [97] pillar_1.8.1           mgcv_1.8-40            survival_3.4-0        
#> [100] KEGGREST_1.34.0        RCurl_1.98-1.8         tibble_3.1.8          
#> [103] crayon_1.5.1           utf8_1.2.2             rmarkdown_2.16        
#> [106] locfit_1.5-9.6         grid_4.1.2             sva_3.42.0            
#> [109] data.table_1.14.2      blob_1.2.3             vegan_2.6-2           
#> [112] digest_0.6.29          xtable_1.8-4           tidyr_1.2.1           
#> [115] stats4_4.1.2           munsell_0.5.0          bslib_0.4.0
```

# Bibliography

Allaire, JJ, Yihui Xie, Jonathan McPherson, Javier Luraschi, Kevin Ushey, Aron Atkins, Hadley Wickham, Joe Cheng, Winston Chang, and Richard Iannone. 2021. *Rmarkdown: Dynamic Documents for r*. https://CRAN.R-project.org/package=rmarkdown.

Auguie, Baptiste. 2017. *gridExtra: Miscellaneous Functions for "Grid" Graphics*. https://CRAN.R-project.org/package=gridExtra.

Bache, Stefan Milton, and Hadley Wickham. 2020. *Magrittr: A Forward-Pipe Operator for r*. https://CRAN.R-project.org/package=magrittr.

Bates, Douglas, Martin Mächler, Ben Bolker, and Steve Walker. 2015. “Fitting Linear Mixed-Effects Models Using lme4.” *Journal of Statistical Software* 67 (1): 1–48. https://doi.org/10.18637/jss.v067.i01.

Bates, Douglas, Martin Maechler, Ben Bolker, and Steven Walker. 2021. *Lme4: Linear Mixed-Effects Models Using Eigen and S4*. https://github.com/lme4/lme4/.

Gagnon-Bartsch, Johann. 2019. *Ruv: Detect and Remove Unwanted Variation Using Negative Controls*. http://www-personal.umich.edu/~johanngb/ruv/.

Gibbons, Sean M., Claire Duvallet, and Eric J. Alm. 2018. “Correcting for Batch Effects in Case-Control Microbiome Studies.” Edited by Morgan Langille. *PLOS Computational Biology* 14 (4): e1006102. https://doi.org/10.1371/journal.pcbi.1006102.

Hornung, R, A-L Boulesteix, and D Causeur. 2016. “Combining Location-and-Scale Batch Effect Adjustment with Data Cleaning by Latent Factor Adjustment.” *BMC Bioinformatics* 17: 27.

Hornung, Roman, and David Causeur. 2016. *Bapred: Batch Effect Removal and Addon Normalization (in Phenotype Prediction Using Gene Data)*. https://CRAN.R-project.org/package=bapred.

Kolde, Raivo. 2019. *Pheatmap: Pretty Heatmaps*. https://CRAN.R-project.org/package=pheatmap.

Kucera, Michal, and Björn Malmgren. 1998. “Logratio Transformation of Compositional Data—a Resolution of the Constant Sum Constraint.” *Marine Micropaleontology* 34 (January): 117–20.

Kuznetsova, Alexandra, Per B. Brockhoff, and Rune H. B. Christensen. 2017. “lmerTest Package: Tests in Linear Mixed Effects Models.” *Journal of Statistical Software* 82 (13): 1–26. https://doi.org/10.18637/jss.v082.i13.

Kuznetsova, Alexandra, Per Bruun Brockhoff, and Rune Haubo Bojesen Christensen. 2020. *lmerTest: Tests in Linear Mixed Effects Models*. https://github.com/runehaubo/lmerTestR.

Leek, Jeffrey T., W. Evan Johnson, Hilary S. Parker, Elana J. Fertig, Andrew E. Jaffe, Yuqing Zhang, John D. Storey, and Leonardo Collado Torres. 2021. *Sva: Surrogate Variable Analysis*.

Maechler, Martin, Peter Rousseeuw, Anja Struyf, and Mia Hubert. 2021. *Cluster: "Finding Groups in Data": Cluster Analysis Extended Rousseeuw Et Al.* https://svn.r-project.org/R-packages/trunk/cluster/.

McMurdie, Paul J., and Susan Holmes. 2013. “Phyloseq: An r Package for Reproducible Interactive Analysis and Graphics of Microbiome Census Data.” *PLoS ONE* 8 (4): e61217. http://dx.plos.org/10.1371/journal.pone.0061217.

McMurdie, Paul J., Susan Holmes, with contributions from Gregory Jordan, and Scott Chamberlain. 2021. *Phyloseq: Handling and Analysis of High-Throughput Microbiome Census Data*. http://dx.plos.org/10.1371/journal.pone.0061217.

Müller, Kirill, and Hadley Wickham. 2021. *Tibble: Simple Data Frames*. https://CRAN.R-project.org/package=tibble.

Nielsen, Torsten O, Rob B West, Sabine C Linn, Orly Alter, Margaret A Knowling, John X O’Connell, Shirley Zhu, et al. 2002. “Molecular Characterisation of Soft Tissue Tumours: A Gene Expression Study.” *The Lancet* 359 (9314): 1301–7. https://doi.org/10.1016/S0140-6736(02)08270-3.

Oksanen, Jari, F. Guillaume Blanchet, Michael Friendly, Roeland Kindt, Pierre Legendre, Dan McGlinn, Peter R. Minchin, et al. 2020. *Vegan: Community Ecology Package*. https://CRAN.R-project.org/package=vegan.

Ritchie, Matthew E, Belinda Phipson, Di Wu, Yifang Hu, Charity W Law, Wei Shi, and Gordon K Smyth. 2015. “limma Powers Differential Expression Analyses for RNA-Sequencing and Microarray Studies.” *Nucleic Acids Research* 43 (7): e47. https://doi.org/10.1093/nar/gkv007.

Simpson, Gavin L. 2019. *Permute: Functions for Generating Restricted Permutations of Data*. https://github.com/gavinsimpson/permute.

Smyth, Gordon, Yifang Hu, Matthew Ritchie, Jeremy Silver, James Wettenhall, Davis McCarthy, Di Wu, et al. 2021. *Limma: Linear Models for Microarray Data*. http://bioinf.wehi.edu.au/limma.

Wickham, Hadley. 2016. *Ggplot2: Elegant Graphics for Data Analysis*. Springer-Verlag New York. https://ggplot2.tidyverse.org.

———. 2021. *Tidyr: Tidy Messy Data*. https://CRAN.R-project.org/package=tidyr.

Wickham, Hadley, Winston Chang, Lionel Henry, Thomas Lin Pedersen, Kohske Takahashi, Claus Wilke, Kara Woo, Hiroaki Yutani, and Dewey Dunnington. 2021. *Ggplot2: Create Elegant Data Visualisations Using the Grammar of Graphics*. https://CRAN.R-project.org/package=ggplot2.

Wickham, Hadley, Romain François, Lionel Henry, and Kirill Müller. 2021. *Dplyr: A Grammar of Data Manipulation*. https://CRAN.R-project.org/package=dplyr.

Wright, Kevin. 2021. *Pals: Color Palettes, Colormaps, and Tools to Evaluate Them*. https://kwstat.github.io/pals/.

Xie, Yihui, J. J. Allaire, and Garrett Grolemund. 2018. *R Markdown: The Definitive Guide*. Boca Raton, Florida: Chapman; Hall/CRC. https://bookdown.org/yihui/rmarkdown.

Xie, Yihui, Christophe Dervieux, and Emily Riederer. 2020. *R Markdown Cookbook*. Boca Raton, Florida: Chapman; Hall/CRC. https://bookdown.org/yihui/rmarkdown-cookbook.
