# Supplementary material for: MBECS: Microbiome Batch Effects Correction Suite
Source: BMC Bioinformatics. 2023 May 3;24:182. doi: 10.1186/s12859-023-05252-w (PMC10155362; doi:10.1186/s12859-023-05252-w)
Supplement: Supplementary file 3 — Additional file 3. Post-correction report. [file 12859_2023_5252_MOESM3_ESM.html]

Post Correction Report


# Post Correction Report

#### MBECS

# Study Summary

A synopsis of covariates, sample dissemination over study
grouping/batches and sample clustering.

## Covariates

A summary of the data-sets covariate information.

```
    sample            group           batch      replicate      sID           
 Length:75          0-0.5:26   09/04/2015: 9   E7aJ40 : 3   Length:75         
 Class :character   1-2  :49   14/04/2016:16   E1aJ40 : 2   Class :character  
 Mode  :character              01/07/2016:21   E6aJ40 : 2   Mode  :character  
                               14/11/2016:17   E7aJ48 : 2                     
                               21/09/2017:12   E7aJ69 : 2                     
                                               E7bJ69 : 2                     
                                               (Other):62
```

## Sample Distribution

How are the samples disseminated over batches and the effect of
interest.

## Sample Separation

The Principal Component Analysis shows sample-relatedness
(clustering) on a 2D plane and can help identify the presence of
confounding factors.

# Visualization

Show how feature abundances are distributed over study grouping and
batches.

## Relative Log Expression (RLE)

Separate samples by covariate of interest (CoI), e.g., treatment or
study group, calculate the median value for every feature count and
subtract it from all samples respectively.

## Heatmap

Show the top ten most dispersed features by interquartile range.

## BOX-plot

Show the top four most dispersed features by interquartile range and
show within batches.

# Variance Assessment

Several different approaches are used to estimate the amount of
variability attributable to covariates of interest.

## Linear Model (LM)

This method fits the linear model ‘y ~ group + batch’ to every
feature respectively and estimates the proportion of variance that the
modeled covariates of interest (coi) account for. The results are
visualized in a box-plot that shows the coi and the residual values.

## Linear (Mixed) Model (LMM)

This method fits the linear mixed model ‘y ~ group + (1|batch)’ to
every feature respectively and estimates the proportion of variance that
the modeled covariates of interest (coi) account for. The results are
visualized in a box-plot that shows the coi and the residual values.

## Redundancy Analysis (pRDA)

A linear regression model is fitted to the feature-matrix
(i.e. counts) while conditioning on one COI at a time to extract the
proportion of explained variance for the variables. Then this procedure
is repeated with switched covariates.

Basically, it takes ‘counts ~ group + Condition(batch)’ and subtract
counts ~ group and see how much variance batch accounts for - then
repeat with group as Condition

## PrincipalVariance Component Analysis (PVCA)

Select the number of principal components that are required to
account for more than 65% of variance and iterate over all PCs and fit a
linear mixed model that contains all covariates as random effect and all
unique interactions between two covariates. Compute variance covariance
components form the resulting model and extract the variance that each
covariate contributes to this particular PC. Standardize variance by
dividing it through the sum of variance for that model. Scale each PCs
results by the proportion this PC accounted for in the first place. And
then do it again by dividing it through the total amount of explained
variance, i.e. the cutoff to select the number of PCs to take, or rather
the actual values for the selected PCs. Finally take the average over
each random variable and interaction term and display in a bar-plot.

## Silhouette Coefficient

Calculate principal components and get sample-wise distances on the
resulting sxPC matrix. Then iterate over all the covariates and
calculate the cluster silhouette. This is essentially either zero, if
the cluster contains only a single element, or it is the distance to the
closest different cluster minus the distance of the sample within its
own cluster divided (scaled) by the maximum distance. Average over each
element in a cluster for all clusters respectively and obtain a
representation of how good the clustering is.
